# The principles of tactical formation identification in association football (soccer) — a survey

**DOI:** 10.3389/fspor.2024.1512386

**Published:** 2025-02-05

**Authors:** Hadi Sotudeh

**Affiliations:** Social Networks Lab, Department of Humanities, Social and Political Sciences, ETH Zürich, Zürich, Switzerland

**Keywords:** football, soccer, formation, shape, position

## Abstract

This paper reviews the principles employed to identify team tactical formations in association football, covering over two decades of research based on event and tracking data. It first defines formations and discusses their history and importance. It then introduces the preprocessing and team/position-level principles. Preprocessing includes match segments and normalized locations followed by data representation using various options, such as average locations, hand-engineered features, and graphs for the team-level and relative locations, distributions, and images for the position-level approaches. Either of them is later followed by applying templates or clustering. Among the limitations for future research to address is the reliance on spatial rather than temporal aggregation, which bases formation identification on newly introduced coordinates that may not be available in raw tracking data. Assuming a fixed number of outfield players (e.g., 10) fails to address scenarios with fewer players due to red cards or injuries. Additionally, accounting for phases of play is crucial to provide more practical context and reduce noise by excluding irrelevant segments, such as set pieces. The existing formation templates do not support arrangments with more or fewer players in each horizontal line (e.g., 6-3-1). On the other hand, clustering forces new observations to be described with previously learned clusters, preventing the possibility of discovering emerging formations. Lastly, alternative evaluation methods should have been explored more rigorously, in the absence of ground truth labels. Overall, this study identifies assumptions, consequences, and drawbacks associated with formation identification principles to structure the body of knowledge and establish a foundation for the future.

## Introduction

The success of the Roman Triplex Acies formation in ancient battles (1) and the power efficiency of migratory birds' V-shaped flight (2) are just two examples that demonstrate the benefits of collective behavior. Formations have also been studied in other domains, including transportation (3), robotics (4), space exploration (5), video games (6), choreography (7), and sports such as American football (8), field hockey (9), handball (10), and association football^1^ (11).

In football, formations have been present since the early versions, as evidenced by available drawings from a festive match played in Italy in 1688, which depict team arrangements on the field, including players' defined distances (12). After the codification of football and its split from rugby in 1863, the first observed formations were 2-2-6, 1-2-7, and 2-3-5 (pyramid). Historically, formations have been modified to balance defensive and offensive capabilities while adapting to rule changes such as offside in 1925. Arsenal's 3-2-2-3 (W-M) from the 1930s, Brazil's 4-2-4 in the 1950s, and the 4-2-3-1 formation used in recent decades are a few examples of this continuous evolution (11) because there is no optimal formation as each has its pros and cons (13, 14).

**Figure 1:**
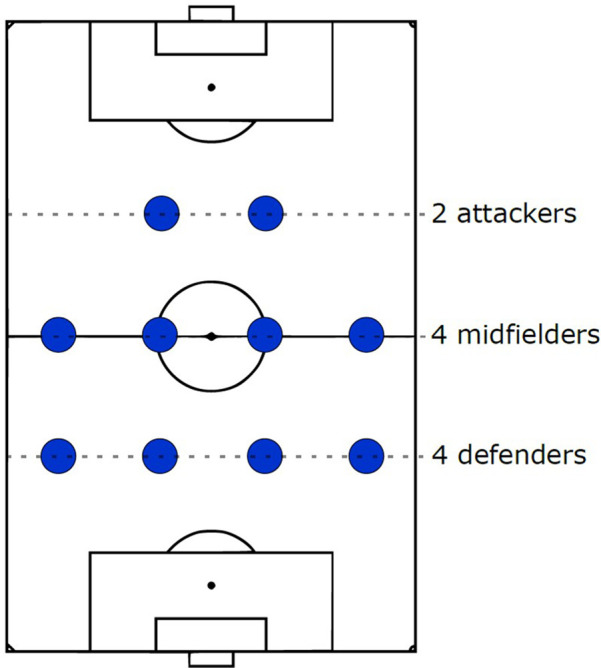
A (symmetric) 4-4-2 formation.

We define “formation”^2^ as an abstraction summarizing each team's spatial arrangement on the pitch over a match using labels (16) that are usually short to communicate useful and relevant information to the target audience in a consistent manner. While this definition means there is no requirement for a standard and unified set of these labels, they are commonly reported using three to five digits denoting the number of outfield players from defense to attack in each horizontal line^3^ usually in a symmetric manner, like 4-4-2 (four defenders, four midfielders, and two attackers), as shown in Figure 1.

Formations can change in a match for various reasons (18) including the match score (19), coach instructions (20), substitutions (21), tactical position^4^ switches, match phases (22–25), opponent (26), mental pressure, injuries, and yellow/red cards. This definition aligns with football as a dynamic interaction process (27) and contrasts with the traditional belief that formations are fixed throughout a match, as reported in “starting formation”^5^ graphics in media and history books (11, 28).

Formations are important to ensure a team operates cohesively, without confusion or delay, while taking advantage of each player's abilities and conserving energy. Therefore, players' confidence is boosted and they can inflict maximum damage on their opponents while remaining less susceptible to attacks (1). Moreover, it serves as a reference (29) for players to remember their organization and responsibilities when distracted (30), helps coaches reduce communication overhead, and shapes the team's collective behavior by creating desired scenarios (31), such as passing options and numerical superiorities. All these reasons could explain why formations are covered in coaching programs, interviews (22, 32–34), training sessions (20), dressing room discussions (35), and media (36).

Formations are also among the first considerations in opposition analysis (13, 20), as highlighted by the *spygate* incident (37). This is because coaches have the freedom to choose any^6^ formation consisting of a goalkeeper and six to ten other starting players to counter opponents (11, 39–41). In addition, there are other factors that can influence a formation choice such as the skills of available players (19), tradition (11), recent results (42), coach and club's principles (43), league (30, 44), home or away (45), and pitch elevation (46).

## Goal

Formation analysis is often carried out qualitatively (47) relying on previous matches using isolated observations (16), most seen arrangements (48), or only out-of-possession moments (49, 50) resulting in a time-consuming and subjective process (51). For instance, comparing the starting formations recorded by two industry data providers for the 2022 Men's World Cup shows only a 65% agreement (52, 53) highlighting the lack of ground truth formation labels (54).

To address these issues, dozens of data-driven studies have been conducted over the past decades to identify formations in a more automated, scalable, and objective manner. These solutions also can have player/coach recruitment in addition to performance and match analysis applications such as studying the relationship between formation choice and various success metrics (e.g., goals, expected goals, scoring zone entries) (30, 55, 56), examining the physical load implications of different formations (57–59), and comparing the identified formations with the instructed ones. Ideally, these approaches, given data availability, can also support real-time applications for media, fans, and specifically the coaching staff to facilitate in-game interventions.

Given the ongoing interest in this problem and the time required to get informed about the relevant developments and their limitations, we recognized the need for a survey on the subject of “formation identification principles in football using event and tracking data” to structure the body of knowledge, prevent redundant efforts, and establish a foundation for future research.

## Method

Our survey is not a systematic review but rather an extensive overview of the principles used to identify football formations^7^ using event and tracking data^8^ in the past decades^9^. We put together similar attempts for each principle found in academic papers, presentations, books, theses, and patents starting with the seminal publications in football and their reference lists. Next, we monitored sources that cited the initial publications and subsequently expanded them to relevant principles from other sports and fields.

In summary, these principles are preprocessing the input data, followed by choosing either the team or position level. Regardless of the choice, there is a data representation and identification step followed up by evaluation. The goal at the team level is to directly report the formation for the entire team while the position level first starts by identifying individual player positions and then maps the set of those positions to a formation label using a pre-defined lookup table. Therefore, this survey also covers tactical position identification methods relevant to formation identification.

**Figure 2:**
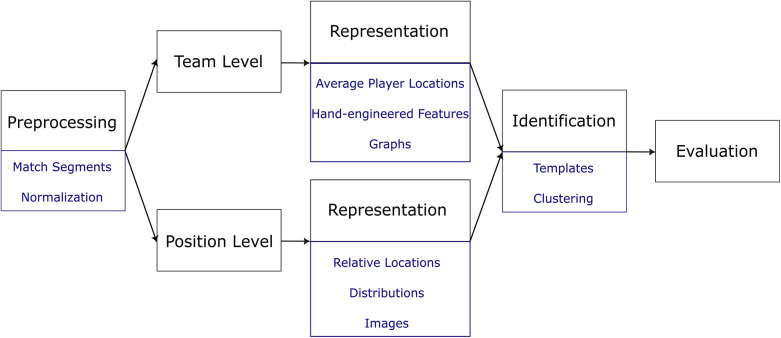
Overview of the formation identification principles.

An overview of these principles and their concepts is depicted in Figure 2. Each step is explained through the remainder sections and subsections of this paper.

## Data

In this section, we introduce the event and tracking data sources. Event data is used only in “Match Segments” while tracking data is employed in all steps shown in Figure 2.

### Event data

The event data commonly includes on-ball actions such as passes, throw-ins, shots, and fouls during a match, often with timestamps, locations, involved players, and other relevant attributes. The collection of event data can be traced back to the 1950s when Charles Reep began recording its basic elements occasionally with pen and paper (69). Today, event data is typically recorded by computer-assisted professional annotators (70).

### Tracking data

The second source is the time series of the ball and player locations obtained through optical tracking cameras installed in the stadiums (71), radar-based systems such as Global Positioning System (GPS) sensors worn by players and inside the ball (72), or computer vision and deep learning models applied to TV footage (73). A tracking dataset with 25 frames per second results in more than three million records per match (74).

## Preprocessing

In this section, the input data is preprocessed by transforming teams to have a consistent attacking direction (e.g., from bottom to top) to negate the effect of half-time side switches, or ignoring the goalkeeper locations, as they may not be relevant. Moreover, the pitch sizes are standardized since they can differ per stadium^10^. The other preprocessing tasks are explained in “match segments” or “normalized locations” subsections.

### Match segments

Since formations can change throughout a match, as mentioned in the introduction, it is necessary to divide the match time into segments, known as phases of play, to report formations. For each phase, coaches instruct their teams to deploy a set of customized principles and arrangements (76, 77). While defining these segments is subjective, there are commonalities among the previous approaches seen in the literature, coaching textbooks, and match reports (78). For example, the England Football Association's training and coaching guide from 1967 introduced the attack (in-possession), defense (out-of-possession), and preparation (transition) phases (77). The transition phase can be divided into attack to defense and vice versa (79). Additionally, set-pieces are considered a separate phase by some coaches because a considerable proportion of goals comes from them (80).

One major difference among these approaches is how the in and out-of-possession phases are divided into smaller sub-phases. For instance, whether to base the division on when each of the opposite team's attack, midfield, and defense lines is broken (81) or to divide the pitch into tactical zones such as the first, middle, and last third of the field (20). This latter approach is reflected in the training grounds of some professional teams to guide player positioning and direction during training sessions (82).

To provide more context, formations should be reported per segment and previous studies operationalized it using a combination of event or tracking data:

1. Fixed time intervals, such as per match half (83) five-minute windows (84), and 15-minute windows subdivided in case of a substitution (85, 86).
2. In and out of possession sequences (25, 87) such as two-minute windows of each separately (88) with tweaks to discard interruptions, short sequences, and some seconds after throw-ins, free kicks, corners, and penalties (89) or consider only sub-windows bigger than five seconds to ignore transitions, and end the time window due to a substitution or half-time break (88).
3. Identification of common in and out-of-possession subphases such as build-up, and low/mid/high blocks using ball zone changes (90) or a Convolutional Neural Network (CNN) trained on labeled tracking data frame visualizations (55).
4. Change point identification by applying g-segmentation on Delaunay adjacency matrices (91), or planarity testing on the graph representation (92) to find distinct intervals (55, 93).

Match segments play a crucial role in identifying formations by excluding segments that have a different nature, such as set pieces. These aspects were overlooked in earlier attempts until recently (55). Additionally, these segments provide more context taking into account the team's arrangement concerning the opponent's influence and ball location, such as build-up (opposed/unopposed) (78). Analyzing segments will also allow one to discuss relevant sub-formations in each phase rather than focusing solely on the overall team arrangement. For instance, it is common to describe a team's build-up as 3–2 (three in the back and two in the middle).

### Normalization

**Figure 3:**
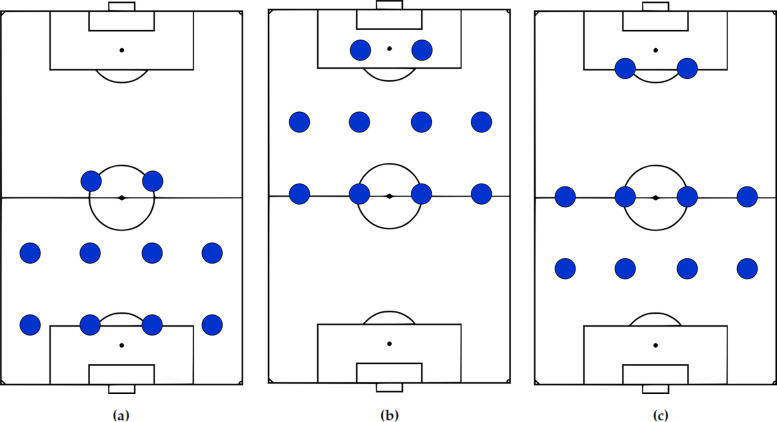
A 4-4-2 in defense **(a)**, attack **(b)**, and with two attackers playing higher up on the pitch **(c)**. All these arrangements should ideally be reported as 4-4-2. While the normalization step can handle **(a)** and **(b)**, it may result in reporting **(c)** as a different formation.

The objective here is to report formations regardless of their on-pitch location (89). For example, Figure 3 illustrates a 4-4-2 formation in various regions and to classify them as the same formation, certain studies have utilized one or both of the following steps, which are part of the Procrustes analysis (94), a statistical shape analysis method with a long history in biology (95).

In translation, the locations of each team's players are relocated with a constant vector (e.g., team centroid or common k-nearest neighbor^11^) to the pitch center (89, 93, 98, 99). To treat compact and narrow formations the same, scaling methods such as min-max (31, 89, 100), scaling to range (101), and division by standard deviation (45, 83, 91, 102–105) are employed.

However, it is crucial to mention that the normalization methods result in unintended transformations of player locations. For instance, applying min-max normalization to an unorthodox 4-4-2, depicted in Figure 3c, where two attackers are located significantly higher up, may not achieve the desired outcome of categorizing it as the same formation as the other 4-4-2 formations shown in Figures 3a,b (106). Therefore, it is desired to achieve the same objective by the other pipeline steps.

## Team level

### Representation

The team-level formation representation should have the following properties:

1. Distinguishing Power: It should differ for distinct formations.
2. Uniqueness: The same formation should have a single and consistent representation.
3. Robustness: Small player location changes that do not alter the formation should not affect the representation.

**Figure 4:**
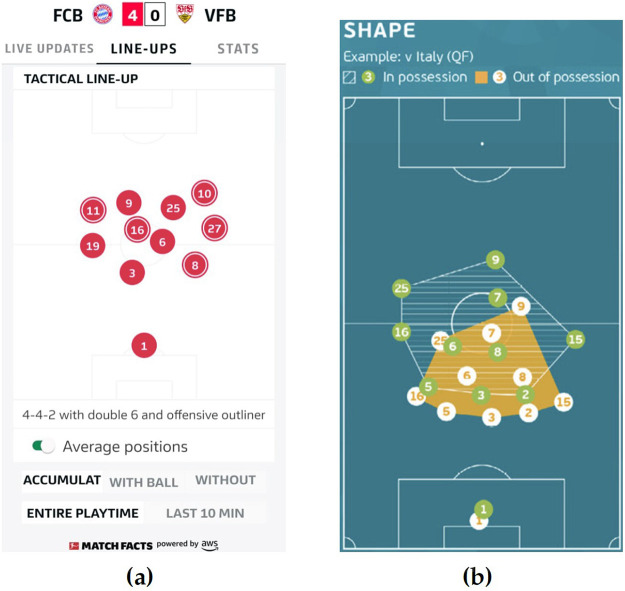
Examples of player average locations seen in the German Bundesliga's official mobile application (112) in **(a)** and UEFA's technical report (113) in **(b)**.

In addition to the raw 2D coordinate vector (107), the following approaches have been proposed:

**Average Player Locations** is the simplest and most common representation (25, 85, 108, 109) in media and reports, as shown in Figure 4. However, a limitation of this representation is that compactness will be interpreted as a direct consequence of averaging. For instance, if a player switches from left to right during the first half, taking average locations per half would locate the player near the pitch center, which is not correct (25, 102) and results in misleading statements (110, 111). One possible mitigation is to compute averages over smaller windows. However, the appropriate time length will depend on the player's position change rate and remains unknown.

**Figure 5:**
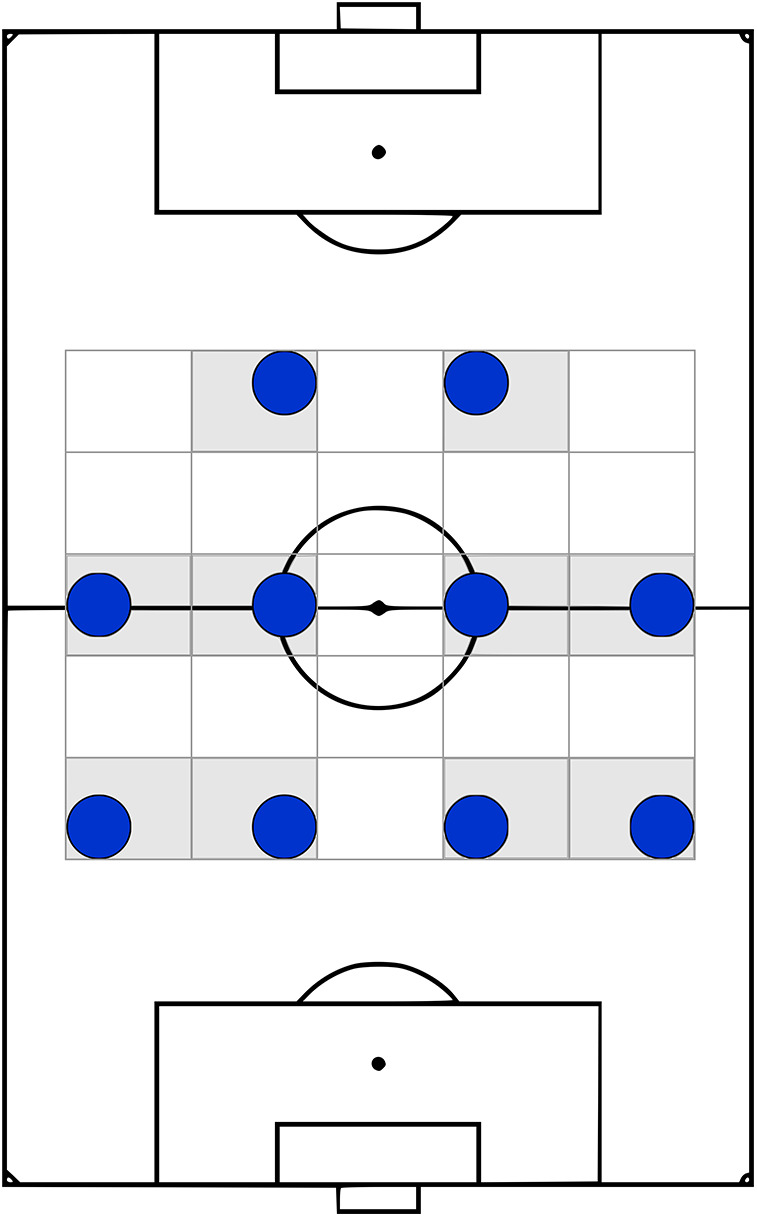
A 5 × 5 grid, inspired by (117), with gray cells indicating the presence of at least one player. This produces a vector of length 25 (5 × 5) to represent the team's arrangement.

**Hand-engineered Features** where relevant indicators for formations such as team centroid, range (83), convex hull, spread, stretch (114), the distance between the farthest players (115), or team heatmaps (116) are computed. For instance, Figure 5 depicts an n×m grid placed around a team, resulting in an nm vector where a cell records the presence or absence of at least one player. The primary burden here remains the identification of relevant features.

**Figure 6:**
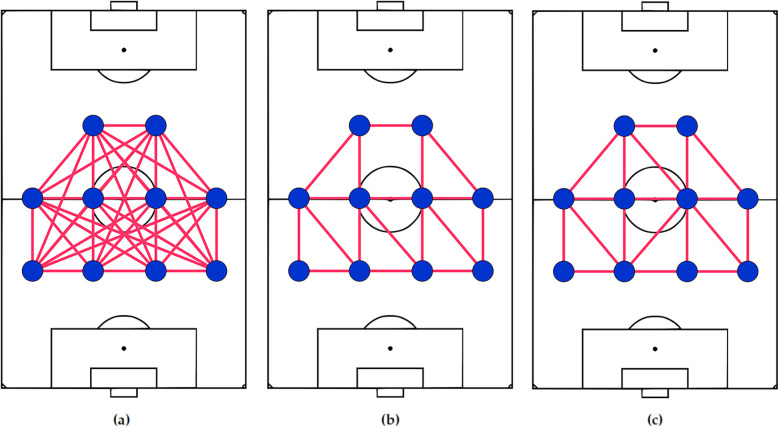
A 4-4-2 representation as a complete undirected graph **(a)**, a union of minimum and second minimum spanning trees **(b)** presented in (82), and delaunay triangulation **(c)** proposed in (104). Considering the properties a team-level representation should have, a complete graph **(a)** can’t distinguish formations since all players are connected. The algorithms producing **(b)** and **(c)** do not guarantee a unique answer and are not robust against small player location changes that don’t affect the team's formation.

**Graphs** representation assumes a set of relations (i.e., edges) among players that can describe their spatial organizations, seen through tracking data, by neighborhood structure rather than aggregated spatial distributions. For a team with *n* players, there are a maximum of n(n−1) directed or n(n−1)/2 undirected relations ignoring self-loops, as shown in Figure 6a (118, 119). Since not all of these relations are relevant, previous studies applied heuristics to well-known graphs, such as minimum spanning trees, nearest-neighbor graphs (10, 92, 120–126), and Delaunay triangulation (DT) (104, 105, 127, 128)^12^ to only consider neighborhood relations. Two examples of them are depicted in Figures 6b,c.

These options have also been successful for similar applications in biometrics such as fingerprint (131–134), palmprint (135), and face identification (136). Additionally, these representations can incorporate inter-team and intra-team relationships when considering both teams together. Coaches have used similar graph representations as a tool for visual communication, too (137).

The primary obstacle lies in identifying the relevant relations. Tactical zones drawn on training grounds serve as just one reference for players to arrange themselves on the pitch and there are other references to consider, such as space (77), ball, goals (77), teammates and opposition players, field markings, nearest players (55), and passing options (77, 121). Moreover, some of these graph-based representations such as DT suffer from (1) a lack of a unique solution and (2) susceptible to minor player location changes, leading to errors in identifying the same formations and inconsistent results.

To the best of our knowledge, previously published formation studies did not consider addressing these two drawbacks when proposing graph-based representations.

### Identification

To assign formations at the team level, both template-based and clustering approaches have been explored, as discussed below. Typically, formations are identified by matching frames or game segments to the most similar template or cluster. A more robust approach, inspired by match analysts' methods and overlooked by previous studies, involves using only frames or segments that exhibit 100% similarity with a template or cluster. Frames that do not fully align can be categorized as transitions, variations, or new formation labels based on similarity scores. Forcing non-perfect matches into predefined templates or clusters will introduce noise and obscure the results.

**Templates** are inspired by common labels like 4-4-2. This option involves preparing a list of formation templates and matching them to the most similar label. The matching process can be accomplished through similarity functions or machine learning algorithms.

Examples of similarity functions are Euclidean-based distances (83, 89, 138), graph edit distance (139), the Freeman code (140, 141), and the sum of element-wise differences divided by the maximal possible distance (84, 142, 143). Machine learning algorithms, such as neural networks, support vector machines, and decision trees, are also employed in some of those attempts (100, 107, 115, 117, 144–150).

One difficulty here is maintaining a consistent and up-to-date list of these templates because of (1) differences across the sources and (2) emergence of new formations over time. For example, Table 1 shows the formations listed by three well-known industry data providers (52, 151, 152). The matching agreement among these providers is just 30% (13 out of 44). This comparison highlights the subjective nature of these labels. Additionally, the FIFA video game series offers 52 formations (153), providing variations to the same label, such as 4-4-2 flat and holding, because players can be arranged in different ways while still using the same label (20).

**Table 1:** Comparison of three data providers’ 44 formations shows 30% agreement (colored rows).

Formation	StatsBomb	Wyscout	Stats Perform
3-1-2-1-1-2	×		
3-1-2-2-2	×		
3-1-4-2	×		×
3-1-5-1			×
3-2-1-2-2	×		
3-2-2-2-1	×		
3-2-3-2	×	×	
3-2-4-1			×
3-3-3-1		×	×
3-3-2-2			×
3-3-1-3			×
3-3-4			×
3-4-1-2	×	×	×
3-4-2-1	×	×	
3-4-3	×	×	×
3-5-1-1	×	×	
3-5-2	×	×	×
3-6-1			×
5-1-2-1-2			×
5-1-2-2			×
5-1-3-1			×
5-1-4			×
5-2-2-1	×		×
5-2-1-2			×
5-2-3			×
5-3-2	×	×	×
5-4-1	×	×	×
4-1-1-3-1	×		
4-1-2-1-2	×		×
4-1-2-2-1	×		
4-1-3-2	×	×	×
4-1-4-1	×	×	×
4-2-1-2-1	×		
4-2-1-3	×	×	
4-2-2-1-1	×		
4-2-2-2	×	×	×
4-2-3-1	×	×	×
4-2-4			×
4-3-2-1	×	×	×
4-3-1-2	×	×	×
4-3-3	×	×	×
4-4-1-1	×	×	
4-4-2	×	×	×
4-5-1	×	×	×

A notable observation about these predefined formation templates is their symmetry, as seen in Figure 1 and coaching documents reported before. However, this assumption appears unrealistic when it comes to player arrangements observed through tracking data.

**Clustering** avoids the difficulties explained in the template-based option and is not restricted to a set of predefined labels. It focuses on learning formations directly from tracking data by inferring the number of players in each horizontal (i.e., defense, midfield, and attack) or vertical (flank) line directly, as shown in Figure 1. Various clustering algorithms, such as complete-linkage (154), K-means (92, 155, 156), Jenks natural breaks optimization & (157), Percentage (101), FOREL (158), and team width/length-based (159), have been proposed to cluster players' x and y coordinates separately per frame. The number of lines can be determined by setting a fixed number (e.g., three) or using optimization methods like the elbow or silhouette method.

## Position level

**Figure 7:**
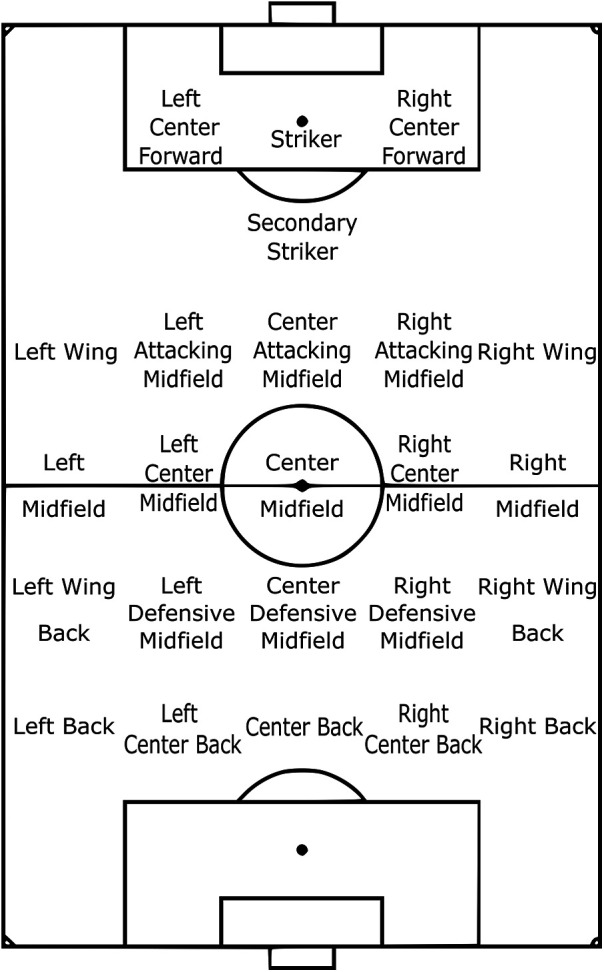
The outfield tactical position locations documented by StatsBomb (52).

Several studies focused on reporting team formations bottom-up by starting from smaller units called positions^13^, which are defined based on where on the pitch players spend most of their match time. Positions are commonly communicated with labels such as center back and right midfield, as shown in Figure 7, for an example.

The reason behind considering positions rather than player identifiers is that players can swap positions, be substituted or sent off during a match, or differ across matches while the set of all possible positions on the pitch remains fixed. Similar to the team-level approach, an appropriate data representation is chosen and later either template or clustering is applied to identify positions. The key assumption employed in the position-level approach is that no two teammates can occupy the same position simultaneously (9). Therefore, a one-to-one mapping is applied to assign either a template or cluster position by solving the assignment problem (161).

Similar to Table 1, we compiled the list of position labels from the same three industry data providers see Table 2 by merging labels with identical descriptions or spatial arrangements on the pitch. This comparison shows a 79% agreement, indicating a stronger consensus than for formations.

**Table 2:** Comparison of three data providers’ 24 outfield positions shows 79% agreement (colored rows).

Position	StatsBomb	Wyscout	Stats perform
Right Back	×	×	×
Right Center Back	×	×	×
Center Back	×	×	×
Left Center Back	×	×	×
Left Back	×	×	×
Right Wing Back	×	×	×
Right Defensive Midfield	×	×	
Center Defensive Midfield	×	×	
Left Defensive Midfield	×	×	
Left Wing Back	×	×	×
Right Midfield	×	×	×
Right Center Midfield	×	×	×
Center Midfield	×		×
Left Center Midfield	×	×	×
Left Midfield	×	×	×
Right Wing	×	×	×
Right Attacking Midfield	×	×	×
Center Attacking Midfield	×	×	×
Left Attacking Midfield	×	×	×
Left Wing	×	×	×
Secondary Striker	×		×
Right Center Forward	×	×	×
Striker	×	×	×
Left Center Forward	×	×	×

### Representation

**Figure 8:**
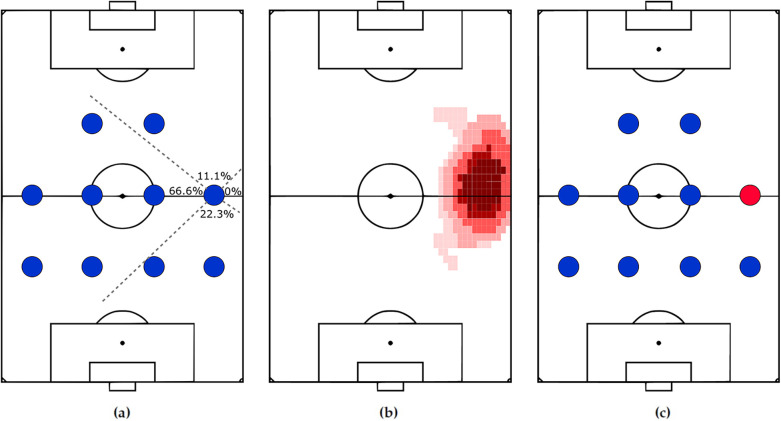
A right midfielders’ representation using relative locations **(a)**, heatmap **(b)**, and color-coded image **(c)**.

Player position data representation proposals apart from the 2D coordinate vectors can be classified into the following categories:

**Relative Locations** are based on how position labels have been named relative to each other. For instance, a left back in a 4-4-2 formation is located to the left of the center backs (45). This approach describes a position using statistics relative to the other players (8) such as the percentage of teammates located in the front, behind, right, and left angle bins (83), as depicted in Figure 8a, the division into 16 instead of four (162, 163), or the amount of created angles (50, 164).

**Distributions** such as bivariate normal distributions (88) and normalized heatmaps containing players' occupancy probabilities (83, 165), as shown in Figure 8b.

**Images** can capture a position's spatial arrangement, as proposed in (99) and shown in Figure 8c to serve as input for image classifiers.

### Identification

Similar to the team level, the position-level identification approaches are templates and clustering.

**Templates** ensure adherence to common position labels. This approach assigns the representation to a predefined set of position templates using one of the following methods:

1. **Rule-based** such as defining arbitrary pitch regions (home areas) for each position. When a player moves outside the designated area, the position is updated accordingly (166, 167).
2. **Similarity functions** such as Chi-square distance for the relative locations representation and naive Bayes as a distance function on the log probabilities of the heatmaps (83).
3. **Machine learning algorithms** such as ResNet on images of color-coded positions, see Figure 8c (99).

The issues discussed for the template-based approach at the team level are also valid here.

**Clustering** moves away from the template issues and various clustering algorithms (78) such as k-means (9, 31, 45, 51, 83, 87, 102, 168–171), Gaussian mixture models (25, 103, 172–175), and hierarchical agglomerative (25, 55, 88, 91, 96, 97, 104, 175–179) have been applied. To determine the number of position clusters, different numbers of clusters (87), dendrogram (88, 105), or a combination of them along with video/match analysts' inputs were considered (55).

## Evaluation

Regardless of the approach, previous studies have generally fallen short in terms of reporting their accuracy, execution time, and required storage. This is understandable given the variations in validation datasets, evaluation metrics, labeling quality, granularity, and expert interpretations (106).

While quantitative evaluation in this area remains difficult due to the lack of ground truth in sports analytics (180), there are other aspects to an evaluation, as suggested for mathematical models in general and sports analytics ones in particular (181, 182). We divide them into design and qualitative categories.

In design, aspects such as realistic assumptions, output robustness to small input data changes, output stability over time, reproducibility, and interpretability can be covered. In the qualitative category, one can address whether the outputs behave as expected in known and boundary scenarios and if the results are intuitive, insightful, and actionable for practitioners (183).

## Discussion & conclusion

While the definition of formations remains an ill-defined problem, we aimed to provide more clarity by defining them as the spatial arrangement of players on the field. Our paper offers an overview of more than 20 years of research on team tactical formations starting from the late 1990s in simulated robotic soccer and American football. The importance of formations is highlighted through opposition analysis, training sessions, and media coverage and the formation identification still is carried out qualitatively to a large extent by counting the number of players in each horizontal line overlooking the vertical disposition.

The main principles were structured as first preprocessing and later taking either a team or position-level approach. The two main concepts employed in the preprocessing step were match segments and normalized locations. The objective of dividing the match time into smaller windows, known as phases of play, is to move beyond reporting one fixed formation for the entire match. Normalized locations aimed to report the same formation for the same arrangements, regardless of where they occurred. However, the potential unintended consequences were not fully understood. Moreover, the same objective can be achieved through other steps of the pipeline without the need for normalization.

After preprocessing, two different paths were followed: The team-level approach looks at a whole team at once while the position level starts with positions as smaller units to build on. In both, the first step is data representation and later, the detection using either qualitatively labeled data (templates) or clustering methods.

Among the data representation options, average locations were the simplest and most commonly used. However, they lead to misleading statements due to the natural outcome of compactness resulting from averaging. When utilizing hand-engineered features or graph representations, it is crucial to carefully select the elements to include in those representations. These elements should align with the references coaches use to instruct team arrangements. Additionally, the representation should be unique for the same arrangements, or arrangements that are not distinguishable due to small player location differences.

After data representation in the team or position levels, formation identification has been achieved by employing domain knowledge through templates or relying on data through clustering. While templates are relatable to public understanding and can be widely accepted, preparing a list of labels and qualitatively assessing them could be cumbersome, especially since there is no worldwide consensus and they change over time. This could be why some adopted clustering to bypass the issues associated with templates. Clustering avoids these issues but on the other hand, requires tracking data of a large number of matches and will limit the future observations to be mapped to one of the existing formation clusters seen in the selected set of matches.

Since our comparison has shown more consensus in position labels than formations, we suggest carefully considering match segments and choosing the position-level approach. For data representation, a graph choice seems reasonable because it can achieve the objectives of the normalization step without facing its drawbacks. When deciding between templates or clustering, it is important to consider the drawbacks of each.

The limitations identified in each step were documented in their respective sections and Table 3 highlights the major ones. Future research can address these limitations and then provide the most value by reporting identified formations and player tactical positions over match time, incorporating contextual factors such as phases of play, substitutions, red cards, scoreline, halftime, and stoppage breaks to reveal formation and position dynamics. Finally, large-scale studies could identify patterns across leagues, seasons, coaches, and teams, as well as how formations counter each other, considering relevant success factors. These advancements will also significantly influence sports science studies that focus on physical load monitoring.

**Table 3:** Some recognized limitations of previous studies.

Limitation	Description
Spatial Aggregation	Introduces coordinates not present in tracking data, as noted in normalization.
Ignoring Phases of Play	Mixes irrelevant coordinates, such as those from set pieces, into results.
Fixed Number of Players	Does not account for scenarios with fewer players due to suspensions or injuries (184).
Existing Pre-defined Templates	Lack flexibility for formations with fewer or more players in each horizontal line (e.g., 6-3-1), see Table 1.
Clustering	Requires extensive tracking data and constrains new observations to predefined clusters, failing to recognize emerging formations.
Forced matching	Assigns a formation to each match frame by selecting the most similar (lowest distance) template or cluster. Instead, one could adopt the approach of match analysts, who focus only on moments with 100% similarity to a formation template or cluster and consider all others as transitions.
Evaluation	Usually is neglected or limited to accuracy-related metrics with an insufficient number of classes. However, those are not applicable in this context due to the lack of ground truth labels (54) and alternative methods, outlined in the evaluation section, should be considered.

## Footnotes

^1^Association/European football or soccer from hereafter is just referred to it as “football”.

^2^The same term has also been used to describe the selection of the best team under specific constraints (15), which is not the subject of this paper. Therefore, we used “tactical formation” in the title to avoid this confusion. In this context, tactical does not mean intended formations but observed ones through data. Hereafter, we will refer to it simply as “formation”.

^3^One can find exceptions where the emphasis is given to the vertical lines, as seen in 2-7-2 denoting the number of players from left to right (17). The digits in this case sum up to 11 as the goalkeeper is also considered.

^4^The term "tactical position", often communicated with labels such as center back and right midfield, typically refers to where players spend most of their match time on the pitch. Since "position" is also used in the literature for player locations (coordinates) from tracking data, we added the adjective "tactical" to avoid confusion.

^5^These graphics are analysts’ educated guesses based on the starting players’ list in addition to players’ tactical positions and team formations from previous matches, as the team officials do not announce their formation.

^6^There is no restriction on the formation choice in the *Laws of the Game* (38).

^7^Excluding studies focused on specific team segments, like defenders (60, 61).

^8^Excluding studies that relied on direct video or image analysis (62–65), as well as partial TV broadcast tracking data (66) because recent advances have allowed for generating full tracking data (67).

^9^The earliest attempts we found date back to the late 1990s in RoboCup and American football (8, 68).

^10^However, the common pitch standardization methods result in distorted player locations (75).

^11^Inspired by players’ alignments with nearest teammates (88, 96, 97).

^12^In which players in adjacent Voronoi cells (“dominant regions”) are connected (129, 130).

^13^Some use the term “role“ to refer to the position (87, 91, 160).
